# Results of Some Statistical and Physiological Researches on Twins

**Published:** 1856-03

**Authors:** 


*Results of some Statistical and Physiological Researches on Twins.*

At a meeting of the French Academy of Sciences, Nov. 26, Mr. Baillarger read a paper containing the results of some statistical and physiological researches on twins. We have thought the subject presents some features which might interest the readers of the Examiner.

"Numerical distribution and relative proportion of the sexes in twin births; hereditary influence.

"I. The facts group themselves into three categories :—

"The first, two boys at a birth.

"The second, two girls at a birth.

"The third, a boy and a girl.

"The result obtained in computing 256 double births shows:—

| | |
|---|---|
| Two boys in . . . . . . | 100 cases. |
| Two girls in . . . . . . | 58 " |
| One boy and one girl in . . . | 98 " |

"It would seem as if the presence of two boys in twin pregnancies is almost twice as frequent as that of two girls. And also that the third class, that of the presence of the two sexes, is almost equal to the first.

"II. The solution of the second question, viz: the relative proportion follows from the above figures.

In 512 twin children are found: Girls, 214 ; Boys, 298.

"The number of boys exceeds that of girls, therefore, by more than one third. This result will certainly seem remarkable if we bear in mind that the proportion of the sexes in the totality of ordinary births, is of 16 girls for 17 boys. So that the difference is in the one case more than a third, and in the other, only a sixteenth. The relative proportion of the two sexes is governed then in twin pregnancies by special laws, quite distinct from those which govern normal births. This fact, interesting in itself, becomes still more so when compared with the documents already collected by M. Flourens on the proportion of the sexes in animals, in which the predominance of males over females is one sixth instead of one sixteenth. I would connect the great predominance of the male sex in twin births with another fact, which is deduced from the general statistics of births, and which at first sight may seem strange. I refer to the far greater proportion of boys among still-born children. This amounts to 17 boys to 12 girls. This singular predominance of boys among still-born children can, in my opinion, be in part if not fully accounted for by the excess of the male sex in twin births, which furnish, as is well known, a pretty considerable contingent to the statistics of still-born children.

"III. Twin births are hereditary in certain families, but in different degrees and different conditions. A large number of facts show, that the daughters of mothers who have had twin pregnancies, have often themselves two children at a birth. This disposition occasionally passes over one generation, when the grand-daughter instead of the daughter has one or several double pregnancies.

"The facts which I have collected would seem to prove that this hereditary disposition is transmitted also through the male. Some men would thus have the faculty of procreating two children at once, although no such hereditary disposition existed in their wives. This fact would have a great physiological importance, and I admit that it should be based on indisputable proofs. I merely indicate it now, and will return to it in a future paper.

"Before closing, it may not be improper to call attention to the fact, that the hereditary disposition of which I have been treating, seems to have been taken advantage of to obtain among animals, species which procreate two young instead of one. Flocks of sheep have thus been formed consisting of individuals which normally bear two lambs. Single births among them become the exception instead of the rule. I have seen a flock composed of nearly one hundred head of sheep, of which each ewe annually brings forth two lambs."

Robert Collins, in his Practical Treatise on Midwifery, gives the result of 16,654 births, occurring in the Dublin Lying-in Hospital, during a period of seven years, from 1826 to 1833. Among these, as appears in a table, pages 164 and following, (Am. Ed.,) there were 240 twin births, in which the sex of the children is mentioned. By a computation of this table, we arrive at results relative to the numerical distribution and proportion of the sexes, materially different from those which Mr.

Baillarger's facts would lead us to expect. Thus in 480 twin children there were : Girls, 234 ; Boys, 246 ; distributed in the following manner :—

| | | |
|---|---|---|
| Two boys in | . . . . . | 73 cases. |
| Two girls in | . . . . . | 67 " |
| One boy and one girl in | . . | 100 " |

By these figures, the presence of two boys in twin pregnancies is only one-eleventh more frequent than that of two girls, and the presence of the two sexes is more than one-fourth more frequent than that of two boys. The number of boys exceeds that of girls by only one-fifteenth, a result but little larger than that obtained from the totality of ordinary births, viz : one-sixteenth.

On the other hand, if we turn to the statistics of the same Hospital, as reported by Alfred H. McClintock and Samuel L. Hardy, for the three years of their connection with the Institution, from Jan. 1st, 1842, to Jan. 1st, 1845, (Practical Observations on Midwifery, p. 329,) we find that during that period, there were 6,634 births, of which 95 were twin births. In these there were : Girls, 79 ; Boys, 111 ; distributed thus :—

| | | |
|---|---|---|
| Two boys in | . . . . . | 38 cases. |
| Two girls in | . . . . . | 22 " |
| One boy and one girl in | . . | 35 " |

Here we are struck with the similarity of the relations existing between the above figures and those which Mr. Baillarger found to exist between the facts collected by him.

The presence of two boys is eight-elevenths more frequent than that of two girls, while in Mr. B.'s cases, it is twenty-one twenty-ninths, or the same thing.

The presence of the two sexes is less than that of two boys, as with Mr. B., though not quite so near being equal.

The number of boys exceeds that of girls more than one-third, as in Mr. B.'s cases, the ratio differing but a unit.

